# Trimester-Specific Diagnostic Accuracy of Amniotic Fluid α-Fetoprotein for Detecting Preterm Premature Rupture of Membranes Versus AmniSure: Short title: AFP Versus AmniSure in PPROM

**DOI:** 10.31661/gmj.v15i.3627

**Published:** 2026-10-04

**Authors:** Homeira Vafaei, Vida Amiri, Naeimehossadat Asmarian, Behrouz Gharesi-Fard, Shaghayegh Moradi Alamdarloo, Nasrin Asadi, Azam Faraji, Shohreh Roozmeh, Khadije Bazrafshan

**Affiliations:** ^1^ Iranian Society of Perinatology, Maternal-Fetal Medicine Research Center, Shiraz University of Medical Sciences, Shiraz, Iran; ^2^ Maternal-Fetal Medicine Research Center, Department of Obstetrics and Gynecology, School of Medicine, Shiraz University of Medical Sciences, Shiraz, Iran; ^3^ Anesthesiology and Critical Care Research Center, Shiraz University of Medical Sciences, Shiraz, Iran; ^4^ Infertility Research Center, School of Medicine, Shiraz University of Medical Sciences, Shiraz, Iran; ^5^ Maternal-Fetal Medicine Research Center, Shiraz University of Medical Sciences, Shiraz, Iran

**Keywords:** Alpha-Fetoprotein, Amniotic Fluid, AmniSure, Pregnancy, Preterm Premature Rupture of Membranes

## Abstract

**Background:**

Preterm premature rupture of membranes (PPROM) is a critical obstetric complication that accounts for 3-10% of pregnancies and a substantial proportion of preterm births, with considerable maternal and neonatal morbidity. Early and precise diagnosis remains challenging because of limitations in current clinical tests, increasing the need for reliable biochemical markers.

**Materials and Methods:**

In this cross-sectional study, 177 pregnant women with clinical suspicion of PPROM before 37 weeks of gestation were enrolled and classified by trimester. Amniotic fluid alpha-fetoprotein (AFP), AmniSure, and Fern tests were performed on vaginal fluid samples. Sensitivity, specificity, and receiver operating characteristic (ROC) curve analyses were used to determine diagnostic performance, and optimal AFP thresholds were established for each trimester.

**Results:**

AFP distinguished PPROM from non-PPROM cases in all trimesters, with area under the ROC curve values exceeding 0.92 and reaching 0.97 in the third trimester. Trimester-specific AFP cutoffs maximized sensitivity (80.5-97.1%) and specificity (83.3–100%). The Fern test showed lower sensitivity and more discrepancies, particularly in early gestation, while the overall agreement between AmniSure and Fern testing was strong (κ=0.85).

**Conclusion:**

Vaginal fluid AFP measurement may be a practical and accurate diagnostic adjunct for PPROM across gestational ages, particularly when trimester-specific cutoffs are applied. AFP testing may be useful where access to proprietary assays is limited, but external validation is required before routine replacement of established diagnostic methods.

## Introduction

Preterm premature rupture of membranes (PPROM) refers to rupture of the fetal membranes before 37 weeks of gestation and is a major obstetric complication, accounting for 3-10% of pregnancies and approximately one-third of preterm births. PPROM is associated with increased short- and long-term maternal and neonatal morbidity and mortality [1]. Although its pathophysiology is multifactorial and includes infection, inflammation, and mechanical factors, the major clinical concern is the risk of ascending intrauterine infection and preterm delivery. Therefore, prompt and accurate diagnosis is essential for optimizing maternal and perinatal outcomes [2, 3]. Diagnosis largely relies on confirmation of amniotic fluid leakage, but detection can be difficult when the clinical presentation is equivocal or classic signs are absent [4]. Bedside tests, including the Fern, Nitrazine, and Pooling tests, are widely used; however, their sensitivity and specificity are imperfect, and their diagnostic yield may vary according to gestational age and sample quality [5, 6, 7]. Approximately 10% of cases may be misdiagnosed even when conventional tests are combined [8, 9]. Therefore, more reliable and accessible diagnostic modalities remain clinically important [1, 3].

Among current tests, the AmniSure assay measures placental alpha microglobulin-1 (PAMG-1) and is widely used as a rapid, noninvasive diagnostic method [10]. Previous studies have reported high sensitivity (95–99%) and specificity (98–100%) for PPROM diagnosis [6, 11]. However, its cost and limited accessibility in some settings have prompted investigation of alternative biomarkers. Alpha-fetoprotein (AFP), a glycoprotein produced by the fetal yolk sac and liver and present in amniotic fluid from early gestation, is normally absent from vaginal fluid in uncomplicated pregnancy; therefore, its detection may indicate membrane rupture [3, 12, 13, 14, 15]. Recent studies suggest that vaginal fluid AFP concentrations increase after PPROM, and several reports support its potential utility as a standalone or adjunctive diagnostic marker [3, 14, 15]. However, evidence remains limited regarding trimester-specific AFP thresholds and direct comparison with established methods such as AmniSure. Head-to-head comparison with conventional methods, including the Fern test, is also clinically relevant because these methods are still widely used [1, 3].

Accordingly, the primary aim of this study was to evaluate the diagnostic accuracy of vaginal fluid AFP for detecting PPROM across the first, second, and third trimesters, using AmniSure as the reference comparator. The secondary aim was to compare diagnostic agreement between AmniSure and the Fern test across gestational age groups.

## Materials and Methods

Study Design: This cross-sectional study was reviewed and approved by the Ethics Committee of Shiraz University of Medical Sciences (approval code: IR.SUMS.MED.REC.1398.238). Pregnant women consecutively referred to Hafez Hospital, Shiraz, Iran, from May 2019 to February 2020 with clinical suspicion of PPROM were screened for eligibility. PPROM diagnosis was based on physical examination, including accumulation of amniotic fluid in the vaginal vault and visible fluid leakage from the cervical os, according to accepted clinical criteria. Written informed consent was obtained from all participants after detailed explanation of the study objectives and procedures. Confidentiality was maintained by anonymizing all data. All procedures complied with the Declaration of Helsinki.

Participants: Pregnant women with singleton pregnancies, suspected PPROM, and gestational age less than 37 weeks were eligible. Women were excluded if they declined to undergo all required diagnostic examinations, if samples were contaminated with blood, or if pregnancy was complicated by congenital anomalies known to increase AFP levels, including neural tube or central nervous system defects. Multiple gestations, pregnancies at 37 weeks of gestation or later, and pregnancies in women with medical comorbidities were also excluded.

Vaginal Sampling and Diagnostic Tests: Participants were categorized into three groups according to gestational age at the onset of suspected PPROM: first trimester (below 16 weeks), second trimester (16-27 weeks), and third trimester (28-36 weeks). All participants underwent AmniSure, Fern, and AFP testing. In each trimester subgroup, women with a negative AmniSure test served as controls. The posterior vaginal fornix was irrigated with 5 mL sterile water. Then, 2–3 mL of fluid was aspirated from the posterior fornix for AmniSure testing, which was used as the reference comparator in this study. An additional 3 mL of fluid was obtained for Fern testing and laboratory AFP measurement. Statistical Analysis: Non-parametric variables were compared using the Mann–Whitney U test and reported as median (Q1-Q3). Receiver operating characteristic (ROC) curve analysis was used to evaluate the diagnostic performance of AFP and to quantify sensitivity and specificity for PPROM diagnosis. Optimal AFP thresholds were determined for each trimester using the Youden index, and an overall threshold was calculated for all gestational ages combined. Inter-test reliability was assessed using Cohen’s kappa coefficient to measure agreement between AmniSure and Fern test results across trimesters. Analyses were performed using MedCalc (version 20.113; MedCalc Software Ltd, Ostend, Belgium) and SPSS (version 26; IBM Corp, Armonk, NY, USA). A P-value <0.05 was considered statistically significant.

## Results

**Table:** Table1. Baseline Characteristics of Pregnant Women with and Without PPROM by Trimester

	PPROM	Non-PPROM	P-value
First Trimester (n=58)	(n=41)	(n=17)	
GA (week)	11 [10-12]	8 [7-11]	0.010*
AFP (ng/mL)	64.88(35.32-78.59)	7(3.2-18.71)	<0.001*
Second Trimester (n=64)	(n=46)	(n=18)	
GA (week)	20.50(16.75-24)	17.50(14.75-20)	0.036*
AFP (ng/mL)	192.54(133.03-211.42)	4.25(1.23-15.48)	<0.001*
Third Trimester (n=55)	(n=35)	(n=20)	
GA (week)	32 [28-35]	30(28-31.75)	0.080
AFP (ng/mL)	313.90(271.83-403.05)	4.25(0.62-8.11)	<0.001*
Total (n=177)	(n=122)	(n=55)	
GA (week)	20(12-27.25)	18 [11-29]	0.637
AFP (ng/mL)	170.04(67.90-258.33)	5(1.50-14.30)	<0.001*

The values are indicated as median (Q1–Q3)

^*^indicates a significant P-value

**Table:** Table2. ROC Analysis of Vaginal Fluid AFP for PPROM Detection Across Pregnancy Trimesters

	Threshold a	Sensitivity	Specificity	AUC	95% CI	P-value
First Trimester	>30.86 (ng/mL)	80.49	100	0.931	(0.833-0.981)	<0.0001*
Second Trimester	>19 (ng/mL)	93.48	83.33	0.925	(0.831-0.976)	<0.0001*
Third Trimester	>23 (ng/mL)	97.14	95	0.971	(0.886-0.998)	<0.0001*
Total	>30.86 (ng/mL)	88.52	92.73	0.925	(0.876-0.959)	<0.0001*

^*^indicates a significant P-value

a: based on the Youden index

**Table:** Table3. Agreement Between AmniSure and Fern Tests for PPROM Diagnosis by Trimester

		Fern test		
	AmniSure test	Yes	No	Kappa Coefficient
First Trimester (n=58)	Yes	36	5	0.808
	No	0	17	
Second Trimester (n=64)	Yes	44	2	0.925
	No	0	18	
Third Trimester (n=55)	Yes	31	4	0.810
	No	1	19	
Total (n=177)	Yes	111	11	0.849
	No	1	54	

The values indicate as frequency

**Figure-1:**
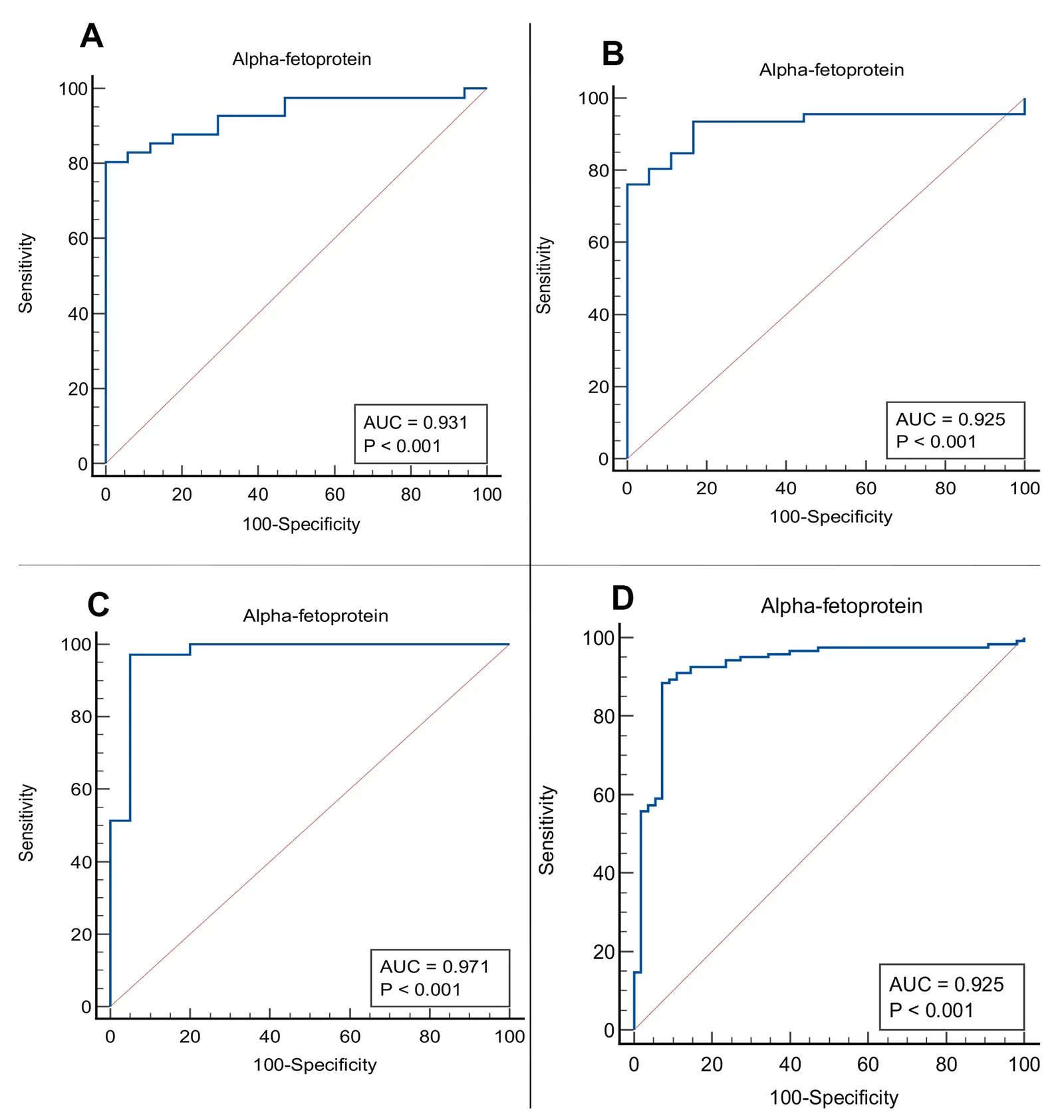
Figure 1. Figure-1. ROC Curves Comparing Diagnostic Performance of Amniotic Fluid α-Fetoprotein and AmniSure Tests for PPROM Across Pregnancy Trimesters. A) ROC Curve for First Trimester of Pregnancy, B) ROC Curve for Second Trimester of Pregnancy, C) ROC Curve for Third Trimester of Pregnancy, D) ROC Curve for Total of cases

In this cross-sectional study, 191 eligible pregnant women were screened. Fourteen declined to participate, resulting in a final study population of 177 participants. Of these, 122 were categorized as PPROM cases and 55 as non-PPROM controls according to AmniSure results. Participants were stratified by gestational age: 58 in the first trimester, 64 in the second trimester, and 55 in the third trimester.

Table1 presents gestational age and vaginal fluid AFP levels among pregnant women with and without PPROM, stratified by trimester. PPROM cases had significantly higher AFP levels than controls in all trimester groups (P<0.001).

Using AmniSure-based classification, AFP levels showed a clear separation between PPROM and non-PPROM groups, supporting its potential role as an accessible adjunctive diagnostic marker.

Table2 summarizes the ROC analysis of vaginal fluid AFP for PPROM detection across pregnancy trimesters.

In the first trimester, an AFP threshold >30.86 ng/mL achieved 80.49% sensitivity and 100% specificity, with an AUC of 0.931 (95% CI: 0.833–0.981; P<0.001). In the second trimester, the optimal threshold was >19 ng/mL, yielding 93.48% sensitivity, 83.33% specificity, and an AUC of 0.925 (95% CI: 0.831–0.976; P<0.001).

In the third trimester, a threshold >23 ng/mL demonstrated 97.14% sensitivity, 95% specificity, and an AUC of 0.971 (95% CI: 0.886–0.998; P<0.001). For all trimesters combined, the threshold >30.86 ng/mL yielded 88.52% sensitivity, 92.73% specificity, and an AUC of 0.925 (95% CI: 0.876–0.959; P<0.001). These findings indicate high diagnostic accuracy of vaginal fluid AFP for PPROM detection across gestational ages, with the best performance observed in the third trimester. The variation in optimal thresholds supports the use of trimester-specific interpretation.

Figure-1 shows the ROC curves for vaginal fluid AFP in the first, second, and third trimesters and in the overall study population.

The ROC curves demonstrate excellent discrimination between PPROM and non-PPROM groups in all gestational age strata. The AUC was 0.931 in the first trimester, 0.925 in the second trimester, 0.971 in the third trimester, and 0.925 overall.

Overall, the ROC findings support the diagnostic value of vaginal fluid AFP for PPROM, particularly when trimester-specific thresholds are applied.

Table3 compares diagnostic agreement between AmniSure and Fern testing for PPROM detection across pregnancy trimesters.

In the first trimester, an AFP threshold >30.86 ng/mL achieved 80.49% sensitivity and 100% specificity, with an AUC of 0.931 (95% CI: 0.833–0.981; P<0.001). In the second trimester, the optimal threshold was >19 ng/mL, yielding 93.48% sensitivity, 83.33% specificity, and an AUC of 0.925 (95% CI: 0.831–0.976; P<0.001). In the third trimester, a threshold >23 ng/mL demonstrated 97.14% sensitivity, 95% specificity, and an AUC of 0.971 (95% CI: 0.886–0.998; P<0.001). For all trimesters combined, the threshold >30.86 ng/mL yielded 88.52% sensitivity, 92.73% specificity, and an AUC of 0.925 (95% CI: 0.876–0.959; P<0.001).

## Discussion

PPROM is an important obstetric condition associated with substantial maternal and fetal risk. Major complications include preterm labor and delivery, low birth weight, umbilical cord prolapse, neonatal respiratory distress, fetal pneumonia, intrauterine infection, sepsis, placental abruption, and hemorrhage [15].

This study found that vaginal fluid AFP had high diagnostic accuracy for PPROM across all trimesters, with particularly strong performance in the third trimester. AFP levels clearly discriminated between PPROM and non-PPROM groups, with AUC values exceeding 0.92 in all trimesters and reaching 0.971 in the third trimester. These findings extend prior evidence supporting biochemical markers as useful tools for PPROM diagnosis [6, 16].

The findings are consistent with previous reports indicating that AFP detection in vaginal fluid has diagnostic value and may provide an accessible alternative or adjunct to PAMG-1-based assays such as AmniSure [16, 18, 19]. The trimester-specific analysis also showed that optimal AFP cutoffs varied by gestational age, supporting the need for gestational age-adapted interpretation.

AmniSure remained the reference comparator in this study and is well established as a highly accurate test for PPROM diagnosis [1, 11]. However, its relatively high cost and limited availability in some settings highlight the need for lower-cost alternatives, particularly in resource-constrained healthcare environments [6, 17].

The strong agreement between AmniSure and Fern testing (overall κ=0.849) confirms the continued value of conventional testing but also highlights its limitations in equivocal cases. Most discrepant results were AmniSure-positive and Fern-negative, consistent with the lower sensitivity of Fern testing in some clinical contexts, particularly when samples are contaminated or amniotic fluid volume is low [11, 20].

Clinically, AFP testing may be useful as an adjunctive diagnostic tool when AmniSure is unavailable, unaffordable, or inconclusive. However, AFP should be interpreted together with clinical examination and other diagnostic findings, and replacement of established tests should be considered only after external validation of the proposed thresholds.

Further multicenter prospective studies are needed to validate trimester-specific cutoffs, assess turnaround time and cost-effectiveness, and evaluate real-world diagnostic performance in diverse clinical settings.

## Limitations

This study has several limitations. First, it was conducted at a single tertiary center, which may limit generalizability. Second, AmniSure was used as the reference comparator; therefore, direct comparison between AFP and AmniSure may be affected by reference-standard bias. Third, AFP cutoffs were derived from the same dataset and require external validation before routine clinical use. Fourth, trimester-specific subgroup sizes were modest, and blinding of laboratory personnel to other test results was not clearly described.

## Conclusion

Vaginal fluid AFP showed high diagnostic accuracy for PPROM across pregnancy trimesters, with the strongest performance in the third trimester. Trimester-specific thresholds may improve interpretation and support AFP as a practical adjunctive diagnostic test when AmniSure is unavailable or unaffordable. These findings support further multicenter validation before AFP testing is adopted as a routine replacement for established diagnostic methods.

## Conflict of Interest

The authors declare no conflicts of interest.

## Declaration of Generative AI

The authors declare that no artificial intelligence (AI)-assisted tools or services were used during the preparation of this work.
